# Transplantation of modified human adipose derived stromal cells expressing VEGF165 results in more efficient angiogenic response in ischemic skeletal muscle

**DOI:** 10.1186/1479-5876-11-138

**Published:** 2013-06-06

**Authors:** Evgeny K Shevchenko, Pavel I Makarevich, Zoya I Tsokolaeva, Maria A Boldyreva, Veronika Yu Sysoeva, Vsevolod A Tkachuk, Yelena V Parfyonova

**Affiliations:** 1 Laboratory of angiogenesis, Russian Cardiology Research and Production Complex, 3rd Cherepkovskaya 15A, Moscow, 121552, Russia; 2 Lomonosov Moscow State University, Lomonosovskiy av. 31-5, Moscow, 119192, Russia; 3 Laboratory of molecular endocrinology, Russian Cardiology Research and Production Complex, 3rd Cherepkovskaya 15A, Moscow, 121552, Russia

**Keywords:** Therapeutic angiogenesis, Cell therapy, Gene modified cells, Adipose stromal cells, Vascular endothelial growth factor, Adeno-associated virus, Ischemia

## Abstract

**Background:**

Modified cell-based angiogenic therapy has become a promising novel strategy for ischemic heart and limb diseases. Most studies focused on myoblast, endothelial cell progenitors or bone marrow mesenchymal stromal cells transplantation. Yet adipose-derived stromal cells (in contrast to bone marrow) are abundantly available and can be easily harvested during surgery or liposuction. Due to high paracrine activity and availability ADSCs appear to be a preferable cell type for cardiovascular therapy. Still neither genetic modification of human ADSC nor *in vivo* therapeutic potential of modified ADSC have been thoroughly studied. Presented work is sought to evaluate angiogenic efficacy of modified ADSCs transplantation to ischemic tissue.

**Materials and methods:**

Human ADSCs were transduced using recombinant adeno-associated virus (rAAV) serotype 2 encoding human VEGF165. The influence of genetic modification on functional properties of ADSCs and their angiogenic potential in animal models were studied.

**Results:**

We obtained AAV-modified ADSC with substantially increased secretion of VEGF (VEGF-ADSCs). Transduced ADSCs retained their adipogenic and osteogenic differentiation capacities and adhesion properties. The level of angiopoetin-1 mRNA was significantly increased in VEGF-ADSC compared to unmodified cells yet expression of FGF-2, HGF and urokinase did not change. Using matrigel implant model in mice it was shown that VEGF-ADSC substantially stimulated implant vascularization with paralleling increase of capillaries and arterioles. In murine hind limb ischemia test we found significant reperfusion and revascularization after intramuscular transplantation of VEGF-ADSC compared to controls with no evidence of angioma formation.

**Conclusions:**

Transplantation of AAV-VEGF- gene modified hADSC resulted in stronger therapeutic effects in the ischemic skeletal muscle and may be a promising clinical treatment for therapeutic angiogenesis.

## Background

Despite advances in revascularization techniques, the treatment of ischemic heart and limb diseases remains a worldwide problem. Therapeutic angiogenesis represents alternative new strategy for ischemia resolution that utilizes regenerative capacity of human body and stimulates natural process of vessel growth, remodeling and tissue revascularization [1].

Commonly adopted approaches for therapeutic angiogenesis include direct introduction of recombinant growth factors and gene therapy. Yet clinical trials have shown several drawbacks of these modalities. Thus low efficacy of recombinant protein administration is explained by dissemination after injection and rapid degradation of therapeutic agent, which requires multiple and long-term infusions thus leading to tremendous expenses [2,3]. Delivery of cDNA coding angiogenic factors via different expression mammalian vectors (plasmids, recombinant viruses) was found more feasible and allowed to achieve great improvement in some cases yet efficacy was still not high enough especially in double blind placebo controlled trials [4]. Many authors discussed possible reasons of gene therapy low efficacy and most of them are univocal to emphasize transfection efficacy and transient transgene expression after plasmid delivery. This can be circumvented by administration of viral vectors but their use is limited due to possible danger of insertional mutagenesis and immune reactions [5,6].

Recently, autologous transplantation of bone marrow stromal cells or endothelial progenitor cells has been shown to enhance angiogenesis and peripheral blood flow [7-9]. However, the regenerative capacity of these cells decreases with age and in patients with co-morbidities such as diabetes mellitus which reduces efficacy of autologous cell administration. Moreover, limited cell viability after transplantation into ischemic tissues also restricts their angiogenic potential [10-12].

It was shown in several experimental studies that this problem could be circumvented by gene modified cell therapy strategy utilizing stem or progenitor cells overexpressing angiogenic proteins [13,14]. To develop a feasible and potent gene modified cell therapy for ischemic diseases the cells should be both effective and accessible in large numbers as well as the chosen viral vector should be both safe and effective in terms of gene delivery. The majority of experimental studies have evaluated gene modified bone marrow stromal cells or endothelial progenitor cells for ischemia treatment [15-17]. However, cells extracted from bone marrow or peripheral blood after mobilization are available in limited numbers and as for bone marrow cells painful aspiration procedure is required.

In contrast to bone marrow or myoblasts, stromal fraction of adipose tissue contains an abundant population of multipotent stem cells that can be easily harvested in high numbers by minimally invasive surgical techniques [18-21]. These adipose –derived stromal cells (ADSCs) share common properties with bone marrow stromal cells and represent a very convenient object for therapeutic use. However the best development of ADSC for angiogenic therapy still needs to be determined.

As for genetic modification of cells the choice of safe and effective gene transfer vector as well as the appropriate transgene determines the quality and safety of the cell product affecting the efficacy of modified cell based therapy. Recombinant adeno-associated viruses (rAAV) are one of the most promising and versatile tools in this field due to low immunogenicity and high transduction potency *in vitro* in many types of both - dividing and non-dividing mammalian cells. Besides that until now no human disease caused by AAV has been identified [22].

In this study we genetically modified human ADSCs with a key regulator of angiogenesis – VEGF165 [23] via rAAV-transduction and then evaluated effects of rAAV-transduction and VEGF165 overexpression on human ADSC growth, differentiation capacity, adhesion and angiogenic factor expression as well as revascularization and functional improvement after intramuscular injection in a mice hind limb model.

## Methods

### Cell culture

Human embryonic kidney (НЕК-293Т) cell line was purchased from ATCC and cultured in Dulbecco’s modified Eagle’s medium (DMEM) containing 10% fetal bovine serum (FBS) (both Gibco, USA) and 1% antibiotic/antimycotic solution. НЕК-293Т were maintained at <50% confluency at 37°C/5% CO_2_ and passaged using 0.01% EDTA/trypsin solution (Gybco, USA).

Human ADSC were isolated from abdominal subcutaneous adipose tissue harvested during surgical operations from patients at age within 32–60 (mean 49*.*2±9*.*8) years. All donors gave informed consent for harvesting of their adipose tissue. Donors with infectious or systemic diseases or malignancies were not included in the study. Adipose tissue was washed extensively with 2 volumes of phosphate-buffered saline (PBS) and then digested at 37°C for 1 h with equal volumes of collagenase (final concentration 66.7 U/ml, Sigma Aldrich, USA) and dispase (final concentration 10 U/ml, BD, USA). Enzyme activity was neutralized by an equal volume of DMEM/10%FBS and suspension was centrifuged at 200 g for 10 min. Cell pellet was resuspended in DMEM/10%FBS and filtered through 40 μm nylon cell strainer (BD Biosciences, USA). Cells were collected by centrifugation, resuspended in AdvanceStem basal medium for human undifferentiated mesenchymal stem cells (HyClone, USA) containing 10% of Advance stem cell growth supplement (CGS) (HyClone, USA), 1% antibiotic/antimycotic solution (culture medium). Red blood cell lysis step was omitted in this protocol and erythrocytes were removed by routine medium changes. ADSC culture was maintained at sub-confluent levels (*<*80% confluency) at 37°C/5% CO_2_ and passaged using HyQtase (HyClone, USA).

### DNA constructs production of rAAV particles and cell transduction

The Hind3/EcoR1 fragment containing human VEGF165 gene was excised from pcDNA3-hVEGF constructed previously [24]. Subsequently it was cloned together with the “stuffer” EcoR1/Xho1 fragment of pCMV-LUC-KEB (а kind gift from «MonA» LLC, Russia) into pAAV-MCS vector (Stratagene, USA), digested by Hind3 and Xho1 to generate pAAV-VEGF165. The stuffer DNA lacking complete open reading frames and RNA degradation signals was used to increase the size of transgene insert for proper incapsidation of recombinant virus [25]. Restriction enzymes were purchased from Fermentas, Lithuania. All necessary plasmid constructs were amplified in DH5α bacterial cells and subsequently purified using Endofree Plasmid Maxi Kit (Qiagen, Germany).

Generation of recombinant AAV particles was performed in HEK293T cells using AAV Helper-Free System (Stratagene, USA). Small-scale vector preparations were made in 100 mm dishes by cotransfection of HEK293T cells with plasmids pAAV-RC, pHelper (Stratagene, USA) and vector plasmid pAAV-hrGFP (Stratagene, USA) or pAAV-hVEGF to produce GFP or hVEGF coding rAAV respectively. Cells that reached 80% confluency were transfected using calcium-phosphate co-precipitation method with 10 μg of each plasmid per dish. Transfected cultures were maintained for 54 h at 37°C in DMEM supplemented with 10% FBS. Thereafter cells were detached and collected by centrifugation at 200 g for 10 min. Cell pellet was resuspended with 1 ml of PBS (per culture dish) and was subject to four freeze–thaw cycles (liquid nitrogen/37°C water bath) vortexing after each thaw. Cell lysate was then incubated with 50 U/ml of Benzonase (Merck, Germany) at 37°C for 30 min to digest cellular nucleic acids. Cell debris was removed by centrifugation at 5000 g for 25 minutes at room temperature and the supernatant (viral stock) was aliquoted and stored at −70°C until use.

One day prior to transduction human ADSC were seeded into 100 mm plates at concentration of 6×10^5^ cells/dish in culture medium. Thereafter medium was removed, cells were washed twice with PBS and the respective medium without CGS was added together with 1–2 ml of viral lysate per dish. 3 h after infection equal volume of medium containing 20% CGS was added to yield a final concentration of 10% CGS. Infected cells were cultured for 48 h and then the percentage of viable cells was determined by trypan blue exclusion.

### Western blotting and ELISA

Conditioned medium samples were assayed to determine human VEGF165 secretion in ADSC culture. One day prior to conditioning gene modified and untreated ADSC were seeded in 60 mm dishes at density of 10^5^cells/dish. Thereafter cells were washed twice with PBS and then respective CGS-free media was added. After 48 h of incubation at 37°C/5%CO_2_ medium was collected and centrifuged at 300 g for 10 min. Collected supernatant was stored at −70°C or used for enzyme-linked immunosorbent assay (ELISA). VEGF165 concentration in condition media samples was measured using human VEGF Quantikine Kit (R&D Systems, USA) following manufacturer’s protocol. For western blotting analysis conditioned media samples were concentrated up to 60-fold using Amicon Ultra–4 units (10000 kDa, Millipore, USA). Concentrated samples were used for SDS-denaturing electrophoresis in a polyacrilamide gel under non-reducing conditions according to standard procedures. Recombinant human VEGF165 (BD Biosciences, USA) was used as a positive control. Separated proteins were transferred to a PVDF membrane (Millipore, USA) with subsequent staining by monoclonal mouse antibodies against human VEGF165 (BD Biosciences, USA) overnight at 4°C and with secondary polyclonal HRP-conjugated goat anti-mouse IgG antibodies (Jackson ImmunoResearch, USA) for 1 hour at room temperature. Two-component chemiluminescent substrate system ECL™ (Amersham Biosciences, USA) was used for detection.

### ADSC proliferation activity assay

To assess population doubling time (PDT) of gene modified (transduced with rAAV at passage 1) or untreated ADSC (passage 2) seeded on 6-well plates (2 × 10^4^ cells/well). After a 9 day incubation average cell numbers for three wells were obtained using a hemocytometer chamber. PDT was calculated as follows:

$$\text{PDT}=\left(\log2\right)*t/\left(\log\left(\mathrm{Nt}/N0\right)\right)$$

where *t* is period of incubation (hours), *Nt* – endpoint amount of cells*, N0* – initial number of cells.

### ADSC cell cycle stage analysis by flow cytometry

ADSC were harvested by 0.25% trypsin/EDTA solution and then fixed in ice-cold 70% ethanol for 2 h following a 30 min incubation in propidium iodide solution (50 μg/ml) containing 200 μg/ml RNAase А (Invitrogen, USA) and 0.1% Triton X-100. ModFit LT 3.2 software (Verity Software House, USA) was used for analysis of cell distribution over cell cycle stages according to intensity of propidium iodide fluorescence in a wavelength range of 600–625 nm (excitation wavelength - 488 nm). Results are presented as a percentage of cells in S + G2/M stages.

### Apoptosis assay

Analysis of spontaneous apoptosis frequency in ADSC culture was performed using Annexin-V FITC Apoptosis Kit (Invitrogen, USA) according to manufacturer’s protocol.

### Adipogenic, osteogenic and endothelial differentiation of ADSC

Gene modified and untransduced ADSC (passage 3) were seeded in duplicate on 6-well plates and maintained in culture medium to reach approximately 90% confluency. To promote adipogenic and osteogenic differentiation of ADSC Mesenchуmal Stem Cell Adipogenesis Kit and Mesenchymal Stem Cell Osteogenesis Kit (Millipore, USA) were used according the manufacturer’s protocol. For endothelial differentiation ADSC were cultured in EGM-2 medium (Lonza, Switzerland) for 14 days. ADSC seeded at the same density and maintained in standard culture medium were used as negative control.

To confirm adipogenesis intracellular lipid droplets were detected using Oil red O staining reagent (Millipore, USA) 2 weeks after induction. To confirm osteogenesis Alizarine Red C staining was used to detect extracellular matrix mineralization 2 and 3 weeks post induction. Endothelial cells were stained for CD31 and VEGFR2 surface antigens and cell counts were obtained using flow cytometry.

### Cell attachment assay

Human adhesion molecule solutions diluted with PBS to a final concentration of 100 μg/ml for collagen type 1 (IMTEK, Russia), 10 μg/ml for vitronectin (Sigma Aldrich, USA), 50 μg/ml for fibronectin (IMTEK, Russia) and 100 μg/ml for laminin (Sigma Aldrich, USA) were added to corresponding wells of a 96-well plate and incubated at 4°C overnight. Blank wells were left uncoated to determine 100% attachment at three cell concentrations and background binding of crystal violet to plastic. After incubation and aspiration of liquid excess wells were washed by PBS and blocked by 0.2% bovine serum albumin for 60 min at room temperature. Wells for 100% attachment were not blocked. The ADSC, GFP-ADSC, VEGF-ADSC (all at passage 3) cell suspensions with concentration of 5×10^5^cells/ml were prepared in prewarmed DMEM gassed with 5% CO_2_. To estimate 100% attachment, additional cell dilutions to 20, 50, and 100% of the working cell suspension were prepared in the same medium. Cells were incubated at 37°C in a 15 ml polypropylene tube with lid off for 10 min in CO_2_ incubator. Thereafter 50 μl of cell suspension together with an equal volume of PBS was added to each well. After 40 min of incubation nonadherent cells in experimental wells were removed by gently washing the well three times with PBS. Attached cells were formalin-fixed for 30 min at room temperature and washed three times with distilled water. In “20%”, “50%” and “100%” wells cells were fixed immediately after incubation. Fixed cells were stained with 0.1% crystal violet for 30 min at room temperature, washed again with distilled water and air-dried. The dye was solubilized in 10% acetic acid by incubating plate on orbital shaker for 5 min at room temperature. Absorbance at 570 nm was measured using a plate Multiscan Ascent reader (Thermo Fisher Scientific, USA). Data from “20%”, “50%” and “100%” wells was used to determine the value for 100% attachment, and then experimental data was expressed as percentage.

### Flow cytometry

To analyze expression of surface antigens human ADSC were stained by specific antibodies: hVEGFR1/FLT1-PE (R&D Systems, USA), hVEGFR2/KDR-APC (R&D Systems, USA), CD31-FITC (BD Biosciences, USA), CD140B (PDGFRβ)–PE (BD Biosciences, USA). Antigen expression analysis was performed on cell sorter MoFlo (DakoCytomation, Denmark) or flow cytometry scanner BD FACS CantoTM II (BD Pharmingen, USA). 10 000 events were acquired and analyzed for antigen expression.

### Quantitative polymerase chain reaction

Total RNA was isolated and purified using RNase Miniprep Kit (Qiagen, USA) according to manufacturer’s protocol. RNA was used for reverse transcription using RevertAid™ first strand cDNA synthesis kit with oligo (dT) primers (Fermentas, Lithuania). Quantitative polymerase chain reaction (qPCR) was performed using primers specific for human VEGF165, ANGPT1, HGF, FGF2 and PLAU mRNAs. The following primers were used: VEGF165 sense, 5’-CAACATCACCATGCAGATTATGC, antisense, 5’-GCTTTCGTTTTTGCCCCTTTC; ANGPT1 sense, 5’-CTCGCTGCCATTCTGACTCAC, antisense, 5’-GACAGTTGCCATCGTGTTCTG; HGF sense, 5’-AGGGGCACTGTCAATACCATT, antisense, 5’-CGTGAGGATACTGAGAATCCCAA; FGF2 sense, 5’-AAGCGGCTGTACTGCAAAAAC, antisense, 5’-TGAGGGTCGCTCTTCTCCC; PLAU sense, 5’-TCAAAAACCTGCTATGAGGGGA, antisense, 5’-GGGCATGGTACGTTTGCTG, β-actin gene: sense, 5’-CCTGGCACCCAGCACAAT; antisense, 5’-GGGCCGGACTCGTCATAC, GAPDH gene: sense, 5’-TGCACCACCAACTGCTTAGC, antisense, 5’-GGCATGGACTGTGGTCATGAG. Quantitative polymerase chain reaction was performed on iCycler iQ™5 real-time PCR detection system (Bio-Rad, USA). SYBR Green PCR mix kit and custom primers were purchased from Syntol, Russia. PCR conditions were: 50°C (2 min), 95°C (10 min), followed by 40 PCR cycles which included denaturation at 95°C (15 sec), annealing at 61°C (for VEGF, HGF, FGF2, PLAU) and 63°C (for ANGPT1) for 30 sec both, 72°C (extension, 30 sec). A melt-curve analysis immediately followed amplification and comprised of denaturation at 95°C (60 sec), cooling to 60°C and a gradient rise of temperature to 95°C with 0.5°C step and continuous acquisition of fluorescence decline. To further confirm specificity of amplification PCR product was analyzed by electrophoresis on 1% agarose gel in tris-acetate-EDTA buffer.

### Animals

8–10 week-old male BALB/c NUDE mice were purchased from Pushchino nursery (Pushchino, Russia). All experimental procedures were performed according to the “Rules for carrying out experiments using laboratory animals” of Russian Cardiology Research and Production Center.

### Matrigel plug assay

Human ADSC at passage 3 were collected by HyQTase treatment and resuspended in PBS (5×10^6^cells/ml). Volume of 400 μl liquid Matrigel™ matrix (BD Biosciences, USA) was mixed with 100 μl of one of the following: PBS as negative control, 5×10^5^ untreated or gene modified ADSC in PBS. Obtained mixtures were injected subcutaneously into inguinal region of BALB/с NUDE mice (n=40) for subsequent analysis of blood vessel formation and sprouting mediated by paracrine action of cells. Animals were equally divided into 4 groups. Negative control group received matrigel with PBS (“PBS” group) while three experimental groups received injections of matrigel mixed with ADSC, GFP-ADSC or VEGF-ADSC (“ADSC”, “GFP-ADSC”, “VEGF-ADSC” groups). At day 14 animals were sacrificed and matrigel plugs were harvested for subsequent immunostaining.

### Hind limb ischemia model

Ten week-old male BALB/с NUDE mice were anaesthetized by intraperitoneal injection of 0.3 ml of 2.5% avertin. Femoral artery was separated in its distal part and ligated proximal to its popliteal bifurcation (keeping *v. femoralis* and *n. ischiadicus* intact)*.* ADSC, GFP-ADSC or VEGF-ADSC (5×10^5^ cells per animal) were resuspended in 150 μl of PBS, and injected in 3 equally divided doses to *m. tibialis anterior, m. gastrocnemius* and *m. biceps femoris* to generate three experimental animal groups: “GFP-ADSC”, “VEGF-ADSC”, “ADSC” (14 animals per group). PBS (150 μl) was injected in negative control “PBS” group. Blood flow was subsequently measured by laser Doppler imaging.

### Laser doppler imaging

Subcutaneous blood flow was measured on plantar side of hind limb using Laser Doppler Imaging System (Moor, UK). Animals were narcotized by inhalation of 1-2% isoflurane/oxygen mixture. Blood flow was measured immediately after ischemia induction and then every 4 days until day 20. Data from three consecutive measurements obtained within 5 minutes with less than 10% deviation was taken for statistical analysis. To exclude influence of external factors data was presented as mean ratio of blood flow in ischemic to healthy limb of the same animal.

### Muscle explants

*M. tibialis anterior* explant culture was prepared on matrigel according to Jang et al. [26] protocol and cultured in M199 medium (Gibco, USA), containing 2% FBS. At day 3 and 7 medium was collected for determination of human VEGF165 concentration by ELISA.

### Specimen preparation and histological analysis

At designated period (day 20 for muscles, day 14 for matrigel plugs) animals were sacrificed by lethal isoflurane dose followed by cervical dislocation. Afterwards *m. tibialis anterior* or matrigel implants respectively were harvested, embedded in O.C.T. Tissue-Tek media (Sakura, Japan), frozen in liquid nitrogen and stored at −80°C. Serial frozen sections (7 μm thick) were prepared for histological analysis. For immunofluorescent staining sections were formalin-fixed and then immunolabeled with rabbit anti-mouse FITC-SMA (Sigma-Aldrich, USA) and rat anti-mouse CD31 (BD Biosciences, USA) primary antibodies. Secondary antibodies donkey anti-rat IgG/Alexa Fluor 594 (Invitrogen, USA) were used. Cell nuclei were counterstained with DAPI. Stained sections were mounted in aqueous-based medium for subsequent microscopy.

For muscle necrosis analysis we used routine hematoxylin-eosin staining of formalin-fixed muscle sections. Necrotic tissue was defined by loss of fiber morphology, cytoplasm disruption, inflammatory cells infiltration and fibrosis.

Microphotographs were taken with Zeiss Axiovert 200 M fluorescent microscope under 100x magnification in 5-7/plug and 7-11/muscle random fields of view (FOV) on an area that covered ≥80% of the section. Manual capillary (CD31+ structures) and arteriolar (SMA + blood vessels with CD31+ inner layer) counts per FOV were made by two independent persons and used to obtain mean values for section, animal or group followed by statistical analysis.

### Statistical analysis

Results were analyzed in Statsoft Statistica 6.0 (Statsoft, USA). Data is expressed as means±standard deviation. P-value of <0.05 was considered of statistical significance. Statistical data distribution was analyzed using a Shapiro-Wilk criterion and appropriate (Student or Mann–Whitney) method was applied to determine statistical significance.

## Results

### Effective transduction of human ADSC by adeno-associated virus serotype 2

**Figure 1:**
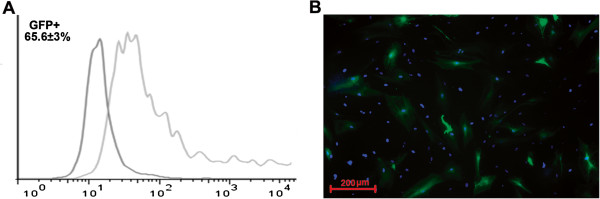
**Human ADSC transduction by recombinant adeno-associated virus. A.** GFP-positive cell count by FACS in GFP-ADSC culture at day 4 after transduction by rAAV. **B.** Representative image of GFP-positive human ADSC (green) transduced by rAAV, 100 × magnification. Cell nuclei are counterstained by DAPI. Data obtained from a total of 10 donors.

Low-passage human ADSC obtained from different donors were transduced using rAAV encoding GFP to assess gene delivery efficacy. Transduced to total cells ratio was counted by flow cytometry. GFP-positive ADSC (GFP-ADSC) were detected as early as day 2 after viral infection. Maximum number of positive cells (65.6±3%) and highest GFP-fluorescence intensity was reached by day 4–5 (Figure 1). GFP signal was detectable for at least 30 days. At day 15 and 30 flow cytometry showed that 45±2% and 25±1.5% of ADSC were GFP-positive respectively. Transduced culture showed about 10% of trypan blue stained cells, indicating moderate effect of rAAV on ADSC viability.

### Increase of VEGF expression and secretion after rAAV transduction of human ADSC

**Figure 2:**
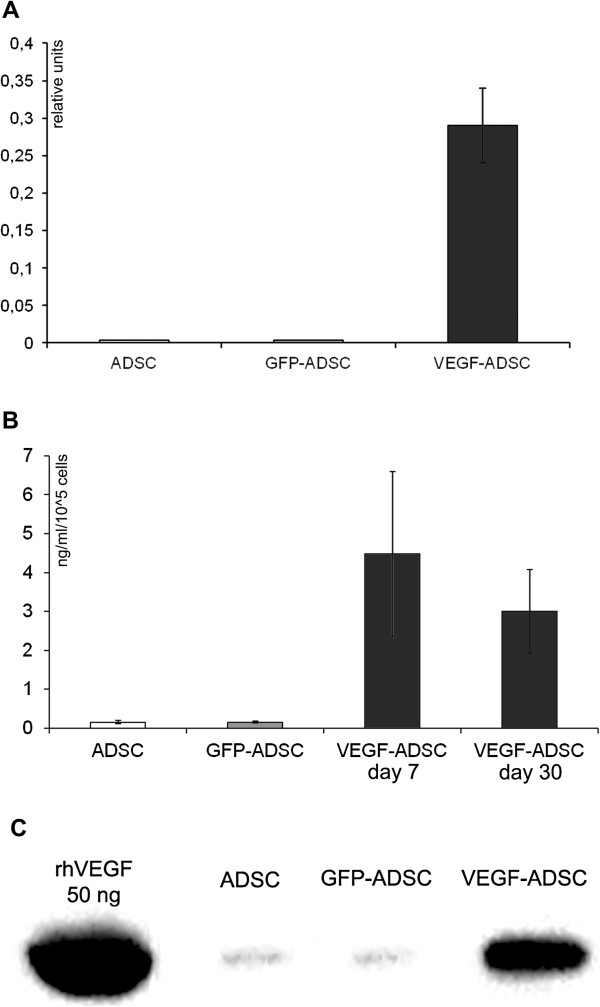
**Validation of VEGF165 expression in AAV-modified VEGF-ADSC. A.** VEGFA expression level in human ADSC 10 days after AAV transduction determined by quantitative PCR. **B, C.** Analysis of VEGF secretion by GFP-ADSC, VEGF-ADSC and unmodified cells using ELISA (**B**) and immunoblotting (**C**). In immunosorbent assay protein content was determined in conditioned media samples obtained at days 7 and 30 post genetic modification of ADSC.

To obtain gene modified ADSC we constructed rAAV vector encoding human VEGF165. In ADSC transduced by rAAV-VEGF (VEGF-ADSC) VEGF165 mRNA level increased 80±15-fold compared to basal expression in unmodified ADSC or GFP-ADSC (Figure 2A). Protein production was analyzed by Western blotting and ELISA. Data presented at Figure 2B, C shows that in VEGF-ADSC secretion of VEGF increased 45-50-fold (4.5±1.8 ng/ml/10^5^ cells) compared to unmodified cells (0.1±0.02 ng/ml/10^5^ cells) or GFP-ADSC (0.09±0.02 ng/ml/10^5^ cells). VEGF concentration in conditioned medium decreased over time during VEGF-ADSC cultivation but remained 30-fold higher (2.9±1.1 ng/ml/10^5^ cells) than in controls (0.09 ± 0.02 ng/ml/10^5^ cells) at day 30 post transduction. Material from a total of 10 donors was used to obtain mean values of VEGF expression increase.

### rAAV-mediated modification of human ADSC suppresses their proliferation activity yet does not influence apoptosis

**Figure 3:**
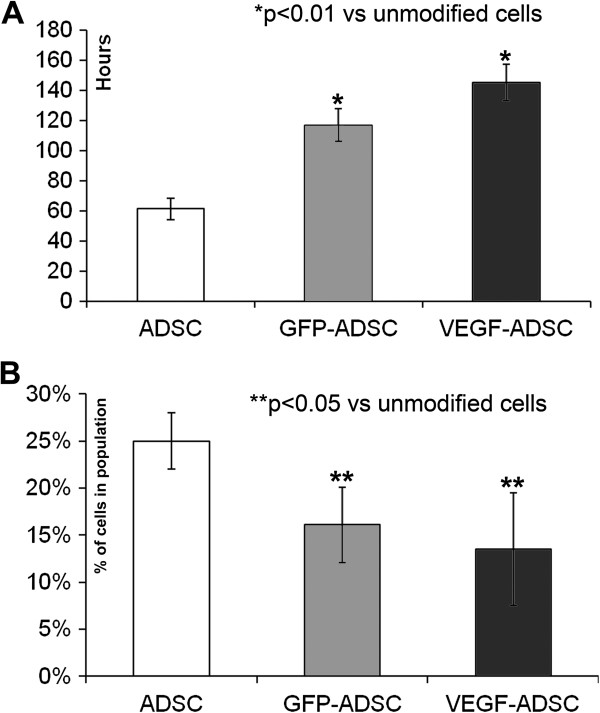
**Proliferation of gene modified ADSC. A.** Population doubling time in GFP-ADSC, VEGF-ADSC and ADSC cultures. Data of five serial runs. **B.** Cells distribution in S-G2 cell cycle stages according to cytometry analysis of GFP-ADSC, VEGF-ADSC and ADSC. Data of three serial runs.

We found that proliferation rate of VEGF-ADSC and GFP-ADSC was reduced compared to unmodified cells (Figure 3A). ADSC population doubling time was 61.3±7 h, while for GFP-ADSC and VEGF-ADSC it was 116.9±11 and 145.4±12 h respectively (n=5, p<0.01 vs unmodified cells). At the same time spontaneous apoptosis rate in all three cell cultures was comparable and comprised about 2±0.5% of total cell population.

Analysis of cell cycle stages distribution in ADSC, GFP-ADSC and VEGF-ADSC cultures (Figure 3B) showed that number of cells in S-G2 stages was more than 1.5-fold lower in modified cells: GFP-ADSC (16±4% cells) and VEGF-ADSC (13±6% cells) compared to unmodified ADSC (25±3% cells; n=3; p<0.05 vs unmodified cells).

### ADSC adhesion does not change after genetic modification

**Figure 4:**
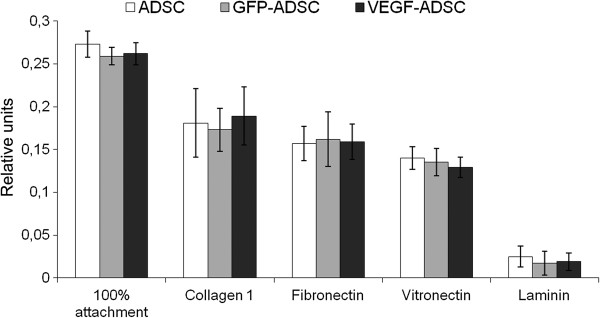
**Data from comparative study of ADSC, GFP-ADSC and VEGF-ADSC adhesion on culture plates coated by collagen 1, vitronectin, fibronectin or laminin (n=4).**

Interactions with extracellular matrix proteins play important role in incorporation and integration to recipient’s tissue, cell viability and their functional properties upon transplantation. ADSC did adhere on main extracellular protein collagen type 1 as well as vitronectin and fibronectin while almost none of cells attached to laminin-coated plastic. We did not observe statistically significant differences in adhesion properties between ADSC, GFP-ADSC and VEGF-ADSC cultures (Figure 4).

### Modified ADSC retain their adipogenic, osteogenic and endothelial differentiation potential *in vitro*

To analyze potential influence of viral transduction and transgene overexpression on differentiation capacity of gene modified cells we performed experiments on adipogenic and osteogenic differentiation of ADSC.

**Figure 5:**
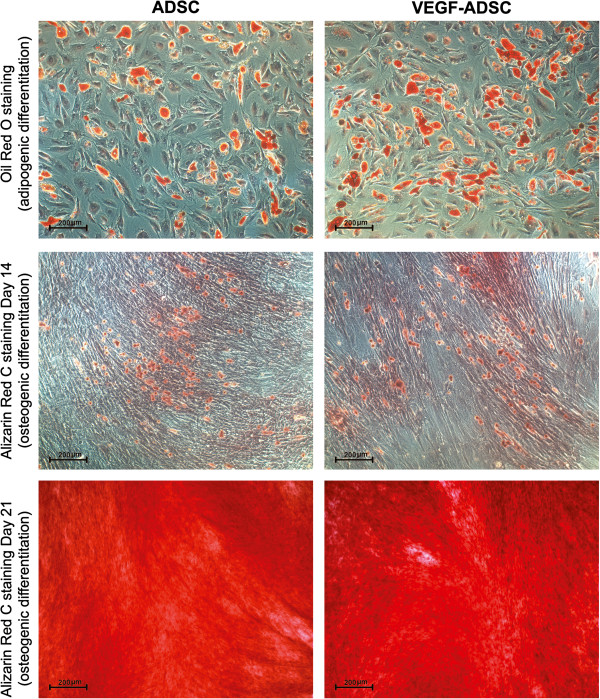
**Adipogenic and osteogenic differentiation of gene modified ADSC.** Representative images of ADSC and VEGF-ADSC cultures stained by Oil Red O (lipid droplets detection, kjadipogenic differentiation, 100 × magnification) and Alizarine Red C (matrix mineralization, osteogenic differentiation, 100 × magnification for “day 14” and 50 × magnification for “day 21”) reagents after incubation in specific differentiation medium, n=3.

Microscopic analysis of gene modified and untreated ADSC stained with Oil Red O reagent after 14 days of incubation in adipogenic media showed >30% of differentiated (visualized by intracellular lipid droplets accumulation) cells (Figure 5). Oil Red O^+^ cell count did not reveal statistically significant differences in both GFP-ADSC (33.7±8.1%) and VEGF-ADSC (34.1±11.5%) as well as unmodified ADSC (34.3±11.7%). Similar results were obtained in osteogenic differentiation assay of ADSC. It was confirmed by Alizarin Red C staining that detects extracellular matrix mineralization. At 14 days of incubation in osteogenic media we detected dye-positive cells in ADSC, GFP-ADSC, VEGF-ADSC culture. At day 21 it was followed by dramatic increase of extracellular matrix calcification in both - modified and untreated cells without significant differences (Figure 5).

Taking into account mitogenic activity of VEGF we analyzed possible effect of genetic modification and VEGF overexpression on endothelial cell fraction in VEGF-ADSC. Using flow cytometry we determined amount of cells that carry CD31 and VEGFR2 endothelial markers in ADSC, GFP-ADSC and VEGF-ADSC (rAAV-modified at passage 1) cultures at passage 2. Less than 1.5% of CD31, VEGFR2-positive cells were detected in all three populations. Subsequently modified and untreated ADSC at passage 2 that reached >90% confluency were subject to incubation in EGM-2 medium to stimulate endothelial differentiation. After 14 days of cultivation in EGM-2 repeated analysis of CD31 and VEGFR2 expression showed that percentage of endothelial marker-positive cells did not change and remained about 1% in all assayed cultures.

### Level of angiopoietin-1 mRNA increases in VEGF-ADSC

**Figure 6:**
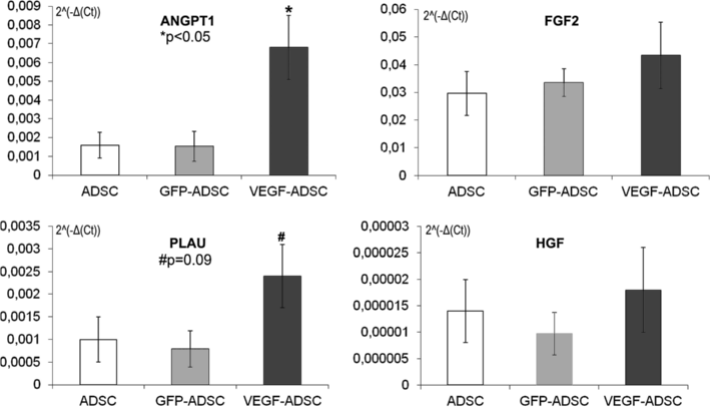
**Comparison of ANGPT1, FGF2, PLAU and HGF genes expression by quantitative PCR in GFP-ADSC, VEGF-ADSC and unmodified ADSC.** Charts represent relative expression for assayed genes from a total of 6 runs.

Using qPCR we studied potential impact of genetic modification and augmented VEGF secretion on expression activity of hepatocyte growth factor (HGF), fibroblast growth factor-2 (FGF2), angiopoietin-1 (ANGPT-1) and urokinase (PLAU) genes in VEGF-ADSC. As shown in Figure 6 we did not find any changes in FGF2 and HGF expression in GFP-ADSC and VEGF-ADSC compared to ADSC. We found a 3-fold increase in urokinase expression in VEGF-ADSC yet it was not statistically significant. At the same time increase of ANGPT-1 expression in VEGF-ADSC was significant and 5.3±0.6-fold higher than in unmodified cells or GFP-ADSC (n=6, p<0.05).

### Analysis of VEGF and PDGF receptors expression on ADSC surface

**Figure 7:**
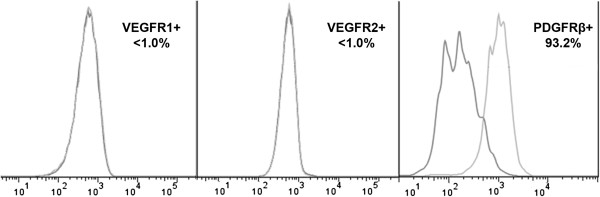
**Analysis of VEGF and PDGF receptors expression on ADSC surface.** VEGFR1, VEGFR2 or PDGFRβ-positive cell count by flow cytometry in ADSC culture.

Analysis of VEGF receptors expression on human ADSC was carried out to assess possible autocrine action of VEGF on VEGF-ADSC functional properties. Flow cytometry of ADSC and VEGF-ADSC (at passage 1–2) from different donors stained for VEGF receptor 1 and 2 showed <1% of positive cells (Figure 7). Taking into account observation of Ball et al. which indicated platelet-derived growth factor receptors (PDGFRα and PDGFRβ) as facultative receptors for VEGF165 [27] we analyzed the presence of cells which expressed PDGFRβ in human ADSC culture. Using specific monoclonal antibodies and subsequent flow cytometry we found that >90% of human ADSC were positive for PDGFRβ (Figure 7).

### Increased vascularisation of matrigel implants after VEGF-ADSC transplantation

**Figure 8:**
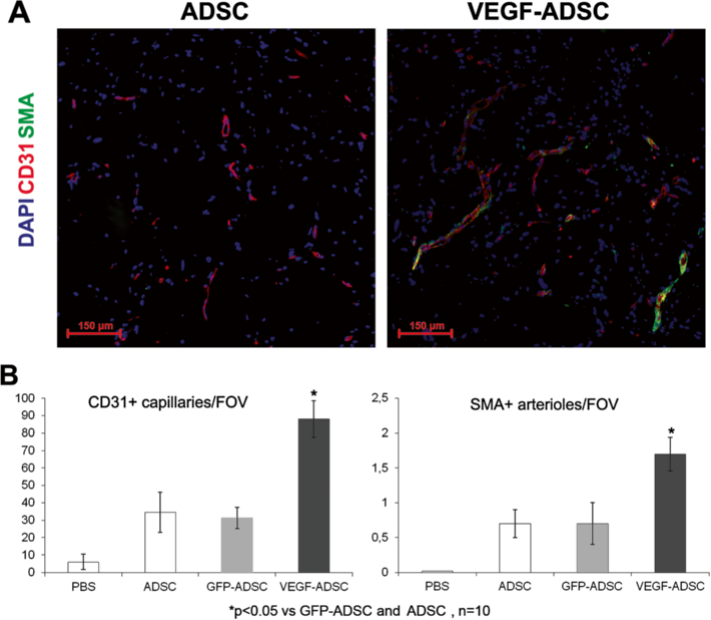
**Effect of VEGF-ADSC or ADSC on vascularization of matrigel implants in nude mice. A.** Representative images of matrigel sections from “VEGF-ADSC” and “ADSC” groups stained by antibodies against murine CD31 and SMA, 100× magnification. **B.** Capillaries and arterioles count in matrigel implants.

We used matrigel plug assay to determine angiogenic properties of gene modified ADSC *in vivo*. At day 14 matrigel implants were harvested and subject to histological analysis (Figure 8). In negative control group we found only small sporadic capillaries (<1 capillary per FOV) were detected while in “ADSC”, “GFP-ADSC” and “VEGF-ADSC” groups formation of vessel network was more evident. Vessel counts revealed a 2.7-fold increase of CD31-positive vessels in group “VEGF-ADSC” (88.1±10.4 vessels per FOV) compared to “GFP-ADSC” (31.3±6.2 vessels per FOV) and “ADSC” (34.5±11.6 per FOV). Number of smooth muscle actin (SMA)-positive vessels was also 2.5-fold higher in “VEGF-ADSC” (1.7±0.24 vessels per FOV) than in “GFP-ADSC” (0.7±0.3 vessels per FOV) and “ADSC” (0.7±0.2 vessels per FOV). Thus capillaries/SMA+vessels ratio did not vary among experimental groups.

### Blood flow recovery after VEGF-ADSC transplantation into ischemic murine limb

**Figure 9:**
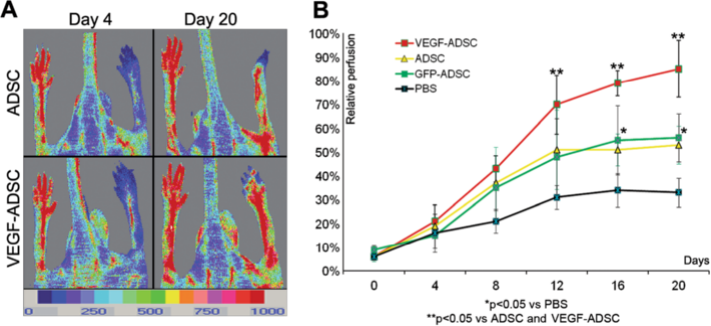
**Reperfusion of murine ischemic limb after ADSC administration. A.** Representative laser-doppler scans of subcutaneous blood flow in mice from “ADSC” and “VEGF-ADSC” groups obtained at days 4 and 20 after ischemia induction and cell transplantation. **B.** Dynamics of blood flow recovery in ischemic limbs within 20 days after intramuscular injection of ADSC, GFP-ADSC, VEGF-ADSC or PBS.

Perfusion assessment in hind limb ischemia model showed maximum blood flow recovery in “VEGF-ADSC” group (Figure 9). By day 20 spontaneous reperfusion of ischemic limb in «PBS» group was feeble and did not exceed 30%. In contrast we observed evident augmentation of blood supply in three experimental groups that received cell injections. At the end of experiment perfusion in “ADSC” and “GFP-ADSC” groups reached 50% and 55% respectively. Blood flow recovery after VEGF-ADSC transplantation was much more effective. At day 12 perfusion in group “VEGF-ADSC” significantly exceeded values in “ADSC” and “GFP-ADSC” by 15-20% and towards the end of experiment (day 20) it reached 80-90%. Thus transplantation of ADSC overexpressing VEGF was more effective than of untreated or GFP-ADSC.

### Transplantation of VEGF-ADSC reduces necrosis and stimulates stable vessel formation in ischemic muscle

**Figure 10:**
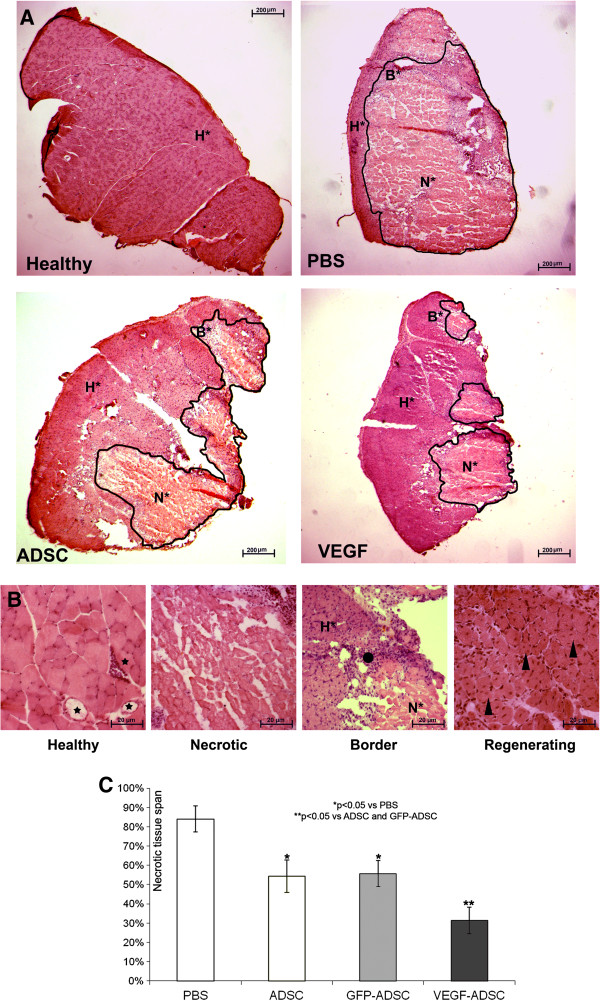
**Morphometric analysis of tissue necrosis in ischemic muscle from study group animals. A.** Images of hematoxylin-eosin stained *m. tibialis anterior* sections. Necrotic tissue is marked by black line. (N* - necrotic tissue, B* - border zone, H* - healthy or regenerating tissue). **B.** Representative images of muscle tissue from different zones of section. Labels: star – vasa in normal muscle tissue with; black dot – inflammatory demarcation zone between anucleic disrupted tissue and regenerating muscle fibers; triangle – regenerating round-shaped muscle fibers with multiple centrally located nuclei. **C.** Statistical data of necrotic tissue area in “PBS”, “ADSC”, “GFP-ADSC” and “VEGF-ADSC” groups. Measurements made in 4–5 animals per group.

Histological analysis of hematoxylin-eosin stained *m. tibialis anterior* specimens obtained at day 20 after and cell transplantation showed significant decrease in necrotic tissue span in «VEGF-ADSC» group (31.3±7%) compared to «ADSC» and «GFP-ADSC» groups (54.3±8.4% and 55.63±6.8%). Animals that received PBS injection as a negative control were characterized by the highest muscle necrosis span that reached 84±6.7% (Figure 10).

**Figure 11:**
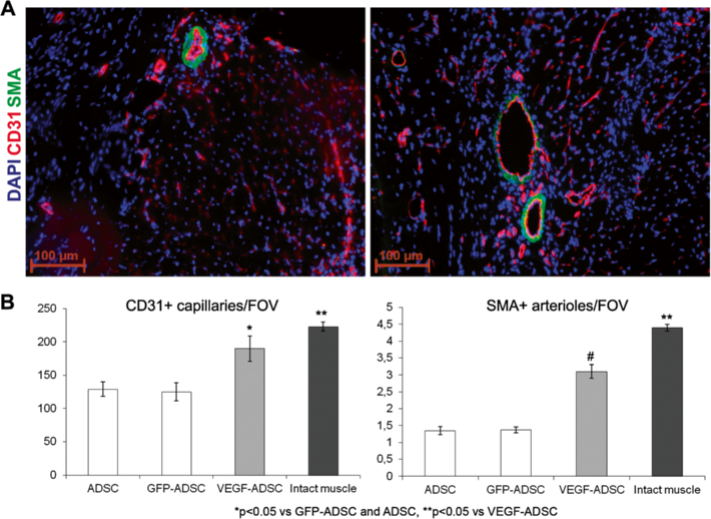
**Vascularization of murine ischemic muscles after ADSC administration. A.** Representative images of *m. tibialis anterior* sections from “VEGF-ADSC” and “ADSC” groups stained by antibodies against murine CD31 and SMA, 100× magnification. **B.** Capillaries and arterioles count in *m. tibialis anterior* sections. Counts made in 5–6 animals per group.

To assess vascular density muscle tissue sections were stained by specific antibodies against mouse CD31 and SMA (Figure 11). Vessel count showed that in “ADSC” and “GFP-ADSC” groups capillary and arteriolar densities were similar reaching 129±11 and 125±14 capillaries/FOV, 1.35±0.12 and 1.37±0.09 arterioles/FOV respectively. In specimens from animals that received VEGF-ADSC capillary density was 189±19 per FOV (p<0.05) with arteriolar density of 3.1±0.2 per FOV (p<0.01). Furthermore, we found that arterioles/CD31+ vessels ratio was similar in all experimental groups and slightly higher in group “VEGF-ADSC” (1% vs 1.6%). In addition morphometric analysis of muscle tissue from group “VEGF-ADSC” did not reveal angioma or abnormal vessel formation.

### ADSC retain viability and transgene expression after transplantation into ischemic muscle

**Figure 12:**
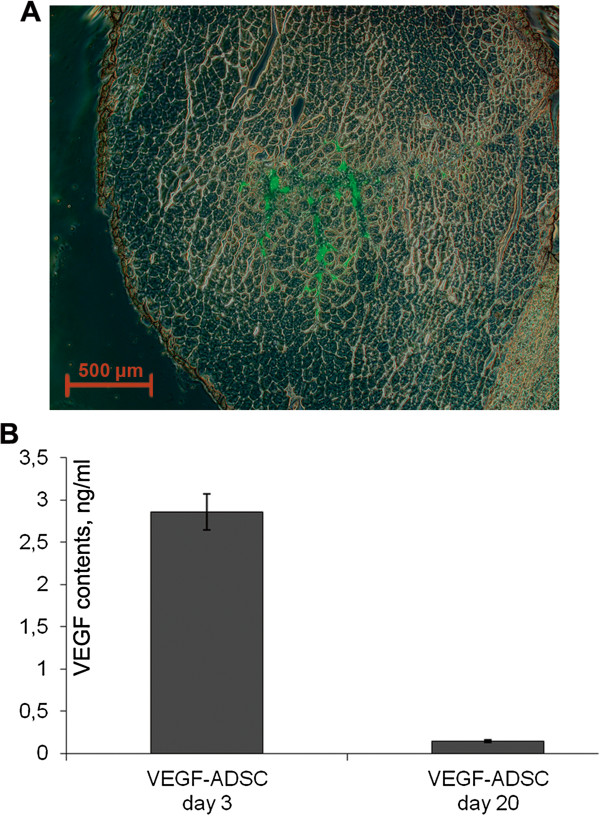
**Human ADSC viability and VEGF expression after transplantation to ischemic murine muscle. A**. Representative image of *m. tibialis anterior* section from “GFP-ADSC” group obtained at day 7 after ischemia induction and GFP-ADSC injection, 50× magnification. GFP-positive cells are distributed in tissue around injection site. **B.** Analysis of VEGF165 content by ELISA in explants culture medium from “ADSC”, “GFP-ADSC”, “VEGF-ADSC” groups obtained at days 3 and 20 after cell trasplantation.

To evaluate viability of transplanted ADSC after injection into ischemic tissue *m. tibialis anterior* specimens from “GFP-ADSC” group were harvested at day 7 after induction of ischemia and cell transplantation. Frozen muscle sections were analyzed using fluorescence microscopy that allowed to detect GFP-positive cells distributed throughout muscle (Figure 12A).

Data from experimental studies indicates that prolonged expression of therapeutic transgene is essential for effective stimulation of angiogenesis and ischemic tissue recovery. Muscle explant model was carried out to confirm the presence of viable and functionally active human ADSC overexpressing VEGF in ischemic muscle at hind limb ischemia experiment endpoint. *M. tibialis anterior* were harvested from “ADSC”, “GFP-ADSC” and “VEGF-ADSC” group animals at day 3 and 20 after cell transplantation and cultured as explant in matrigel. In culture medium samples collected after 3 days of “VEGF-ADSC” explant incubation (obtained at day 3 after cell transplantation) human VEGF165 concentration determined by ELISA reached 2.86±0.21 ng/ml (Figure 12B). Protein concentration was expectedly lower (0.145±0.015 ng/ml) in conditioned medium from muscle explants harvested at day 20. In addition comparison of VEGF concentration in culture medium samples collected at day 3 and 7 post incubation of explant culture revealed accumulation of VEGF. It indirectly confirms presence of functionally active human VEGF-ADSC in ischemic muscle up to 20 days post transplantation. In contrast to “VEGF-ASDC” human VEGF165 concentration in explant cultures from “GFP-ADSC” and “ADSC” groups was below limit of detection.

## Discussion

Gene modified cell-based therapy for ischemic disorders: myocardium infarction and limb ischemia is a rapidly evolving trend in experimental and regenerative medicine. Promoting angiogenesis in ischemic tissues via paracrine action of transplanted modified cells is an emerging alternative modality for patients who are unsuitable for surgical and interventional revascularization. Still choice of appropriate cell type, angiogenic factor and gene delivery tool are crucial issues for efficacy and safety of the method.

Regarding type of cells there are certain issues concerning their derivation and preparation prior to grafting. Thus, embryonic stem cells application is doubtful due to ethical reasons, potential risks of teratogenesis and immune response to their differentiated progenies [28]. Use of endothelial progenitor cells from peripheral blood and bone marrow are limited by expensive procedures of isolation and difficulties in obtaining sufficient amount of cells. Regarding the latter point it is known that prolonged incubation of cells *in vitro* prior to transplantation is associated with potential risks of malignancy, proliferation decrease and commitment to terminal differentiation. Use of skeletal myoblasts or bone marrow derived mesenchymal stromal cells (BMMSC) is associated with painful isolation procedure of muscle biopsy and suprailiac puncture respectively.

ADSC used in our study share a lot of similar properties and characteristics with BMMSC, while they are easier to obtain in sufficient quantity using minimally invasive liposuction procedure. Various data suggests that up to 1.5 × 10^6^ adipose stromal cells can be isolated from 1 ml of adipose tissue [29,30]. This allows to reduce the time of cell propagation *in vitro* prior to transplantation. As for therapeutic angiogenesis, human ADSC produce a wide spectrum of biologically active molecules – angiogenic growth factors, cytokines, proteases etc. [31,32]. Multiple experimental studies accumulate data on relatively high therapeutic potential of ADSC for tissue regeneration and stimulation of angiogenesis [21,33,34]. However well-known reduction of cell regenerative potential with age and among patients with severe co-morbidities is also relevant for ADSC. Donor age-associated decrease of proliferation activity and differentiation capabilities was shown for human ADSC [35,36]. Angiogenic potential of ADSC also decreases with ageing and is characterized by reduced secretion of VEGF, HGF, angiopoietin-1 and other angiogenic factors [37]. Thus, attempts to improve regenerative potential of ADSC are reasonable.

We have shown high efficacy of rAAV-mediated genetic modification of human ADSC. Using rAAV encoding VEGF165 we obtained human ADSC with increased level of VEGF165 secretion which retained for at least 30 days. VEGF-A and particularly its most abundant 165-amino acid isoform triggers multiple reactions promoting new vessel formation and growth [23] that supported our choice of therapeutic gene in presented study. Observed gradual decrease of transgene expression can be attributed to proliferation activity of ADSC together with known episomal subsistence of rAAV [38]. Moreover cellular mechanism of addressed methylation can be activated after transduction leading to suppression of cytomegalovirus promoter which triggers transgene expression in our vector [39].

Potential influence of genetic modification and transgene expression on cell behavior and functional activity is frequently kept out of consideration while this issue is of great importance, especially for potential clinical application. We examined possible effects of rAAV-transduction and VEGF overexpression on functional properties of ADSC which included proliferation, spontaneous apoptosis, adhesion and differentiation capability.

We observed a decline in ADSC proliferation after modification by rAAV that was evident by increase of population doubling time as well as decrease in number of cells in S–G2 stages of cell cycle. At the same time spontaneous apoptosis rate did not exceed 2% in modified and unmodified cells. These results contribute to previously published data that showed transient cell cycle arrest after AAV transduction of embryonic fibroblasts and BMMSC [40]. This effect was observed whenever wild-type, recombinant or genome-empty AAV particles were used. It was suggested that changes in expression profile and decreased proliferation were related to initial stage of virus entry and caused by capsid proteins interaction with cellular signaling pathways [40]. Growth inhibitory effect was transient and proliferation restored to normal level over time of cell passaging [41]. It appears that proliferation decline of rAAV-modified ADSC occurs by a common mechanism.

ADSC are known to be able to differentiate into adipocytes, chondrocytes, osteoblasts, myocytes, neural cells, cardiomyocytes, endothelial and liver cells when cultured in special induction medium [42,43]. Analyzing data from our differentiation experiments we concluded that rAAV-mediated genetic modification of human ADSC and VEGF overexpression did not alter their adipogenic and osteogenic differentiation properties.

There are several observations indicating ability of ADSC for endothelial differentiation [44,45] as well as evidence for presence of small amount of endothelial cells in ADSC population at early passages [18,19]. In our experiments we did not find an increase in amount of cells positive for endothelial markers CD31 and VEGFR2 in VEGF-ADSC compared to unmodified ADSC population. This suggests that VEGF overexpression neither induces endothelial differentiation of modified ADSC nor stimulates proliferation of preexisting endothelial cells in ADSC culture.

Adhesion tests conducted in our study were based on a fact that interaction with extracellular matrix proteins is a key factor that contributes to cell viability and integration into host tissue after transplantation [46]. We found that both modified and untreated ADSC showed very common adhesion on collagen type 1, vitronectin and fibronectin. Thus we can suggest that rAAV-mediated genetic modification did not alter expression of adhesion molecules on cell surface of ADSC. Our results showing low ADSC adhesion on laminin are not surprising taking into account published observations which indicate diminished or lack of α6, α7 and ß1 integrins expression in ADSC-components of α6/ß1 and α7/ß1 receptors for laminin [47,48].

Since VEGF can regulate multiple signaling pathways [23] we next determined whether expression of HGF, FGF2, urokinase and angiopoietin-1 might be altered in VEGF-ADSC. HGF and FGF2 are mitogens and chemoattractants for both endothelial and mural cells and directly participate in angio- and arteriogenesis [4]. Angiopoietin-1 is characterized as a stabilizing factor that provides formation of functionally mature vessel network [49]. Urokinase plasminogen activator is a key regulator of extracellular proteolysis which is responsible for cleavage activation of growth factors and migration of endothelial cells during vessel growth [50,51]. We found almost 3-fold yet not statistically significant increase of urokinase expression while expression of HGF and FGF2 did not change. Another interesting finding is a 5-fold increase of angiopoietin-1 expression in VEGF–ADSC compared to GFP–ADSC or unmodified cells.

We assumed that up-regulation of angiopoietin-1 expression occurs due to autocrine action of VEGF165 produced by VEGF-ADSC. However according to our data supported by other studies [30,52] cultured human ADSC population contains <1% of cells that express receptors to VEGF165 - VEGFR1 and VEGFR2. At the same time we found that >90% of ADSC carry receptor to platelet-derived growth factor - PDGFRβ. There is a published observation that PDGFRα and PDGFRβ can act as a facultative receptor for VEGF [27]. Furthermore it is known that PDGFR activation leads to increase of angiopoietin-1 expression [53]. Considering that more than 90% of human ADSC are PDGFRβ-positive we can speculate that increased expression of angiopoetin-1 in VEGF-ADSC could be attributed to PDGFRβ-mediated autocrine action of VEGF.

In our study we evaluated therapeutic potential of gene modified human ADSC in terms of their ability to induce angiogenesis in ischemic muscle tissue. It was found that matrigel implants after transplantation of VEGF-ADSC had higher vascular density than after delivery of untreated cells or ADSC transduced by a reporter gene. Along with capillary formation we also found proportional increase in amount of mature blood vessels characterized by smooth-muscle wall. This can occur due to the fact that cells transplanted in matrigel produce other angiogenic factors besides VEGF that can promote vessel maturation and stabilization.

Key angiogenic property of cell therapies in experimental study is ability to induce reperfusion of ischemic tissue in appropriate animal models. We used hind limb ischemia model to show that VEGF-ADSC transplantation led to significantly higher perfusion restoration than after untreated of GFP-transduced cell administration. It was also found that intramuscular injection of VEGF-ADSC had a tissue-protective effect and led to vivid decrement of necrosis span. VEGF is known to be significant antiapoptotic factor that can enhance cell survival. We suggest that increased VEGF content during the first days after onset of acute ischemia and cells administration leads to promotion of cell survival and thus to reduction of necrotic disruption in muscle tissue.

We should also point that during the experiment we did not observe any blood flow decrease after cell administration or rapid “plateau” formation like it was previously described for plasmid-mediated gene delivery due to short-term transgene expression [4]. This can be explained by presence of viable and functionally active ADSC that produced VEGF throughout the experiment. In our muscle explant experiments we showed that VEGF-ADSC retain functional activity even at long terms after injection (up to 27 days) and produce VEGF in detectable quantities. Thus we can confidently attribute tissue protection and restoration of blood flow in mice that received VEGF-ADSC to increased long-term VEGF production by modified cells. As for decrease of human VEGF content in murine tissue by day 20 we suggest that cells undergo apoptosis over time. Besides that methylation of CMV promoter which drives VEGF expression in our vector could take place. Taking into account that Nude mice were used we find it hard to assume possible rejection of transplanted cells as far as this animal strain lacks T-cells immunity which plays a crucial role in graft rejection. Still, it seems that produced amount of VEGF is sufficient to trigger angiogenesis and relief tissue ischemia via restoration of blood flow.

Histological analysis of ischemic muscle injected with modified VEGF-ADSC revealed that capillary density was significantly higher than in specimens from animals that received untreated cells or GFP-ADSC. We noticed that this increase was not only due to higher capillary count, but also to SMA-positive blood vessels of arteriolar type. Furthermore arteriole/capillary ratio was constant throughout experimental groups that indicated formation of a stable mature vascular network. Thus, despite high level of VEGF produced by modified ADSC we did not observe any evidence for abnormal tumour-like vascular structures in muscle as it was previously shown e.g. in studies of adenovirus-mediated delivery of VEGF gene [54]. In contrast to matrigel implants experiment in case of skeletal muscle we do not state that increase of vascular density in experimental groups was only due to *de novo* formed vessels. Besides promoting endothelial cell proliferation VEGF also prevents endothelial apoptosis leading to survival of preexisting vessels. There was surely a vast amount of persisted capillaries in the muscles due to VEGF anti-apoptotic effect of VEGF.

It is often speculated that low efficacy reported in clinical trials using gene delivery of VEGF alone can be explained by its high mitogenic activity which is not supported by vessel stabilizing stimuli and consequently ends up with dissociation of formed capillaries [55]. This led to a concept of combined gene delivery indicating that combinations of angiogenic and vascular stabilizing factors should be used to treat ischemic tissues [55-58]. Cell therapy for ischemic disorders has a valuable advantage since transplanted cells produce a whole “cocktail” of biologically active molecules which render combined effect in impaired tissue. We suggest that stable vessel formation observed in our study is mediated by aforementioned ADSC ability to produce a wide spectrum of angiogenic factors including ones responsible for vessel stabilization and maturation: angiopoietin-1, TGF-β, PDGF, which can act synergistically with increased production of VEGF165 by modified cells. Besides that, genetic modification can alter cell’s expression profile. Observed increase in expression of angiopoietin-1 in VEGF-ADSC can further contribute to formation of mature vascular network that also supports therapeutic effect of transplanted cells. Increased concentration of VEGF in ischemic tissue plays a substantial role in vessel stabilization and therapeutic effect if maintained over a significant period of time, which was achieved in our study and exceeded a substantial term of 3 weeks.

## Conclusions

Thus we can conclude that human ADSC with their accessibility and angiogenic paracrine activity is an appropriate and preferable type of cells for therapeutic angiogenesis. Obtained results indicate that relatively safe rAAV holds great potential for gene transfer into human ADSC. Taken together, we suggest that the use of AAV-modified ADSC overexpressing VEGF165 is a feasible and effective approach for stimulation of stable vascular network formation in ischemic muscle and can be implied for therapeutic angiogenesis or tissue-engineered transplants. Further study and improvements in vector design, regulated transgene expression, cell preparation and propagation conditions are still to be completed to allow clinical application of modified cell-based therapeuticals.

## Abbreviations

ADSC: Adipose derived stromal cells; BMMSC: Bone marrow derived mesenchymal stem cells; CGS: Cell growth supplement; DMEM: Dulbecco’s modified Eagle’s medium; ELISA: Enzyme-linked immunosorbent assay; FBS: Fetal bovine serum; FGF2: Fibroblast growth factor 2; FOV: Field of view; GFP: Green fluorescent protein; HEK293T: Human embryonic kidney 293 T; HGF: Hepatocyte growth factor; PDGF: Platelet derived growth factor; PDT: Population doubling time; PBS: Phosphate buffer saline; rAAV: Recombinant adeno-associated virus; SMA: Smooth muscle actin; VEGF: Vascular endothelial growth factor

## Competing interests

The authors declare that they have no competing interests.

## Authors’ contributions

ES performed all experiments, drafted the manuscript and wrote the paper. PM performed animal surgery, carried out Laser Doppler imaging, performed the statistical analysis and wrote the paper. ZT participated in Matrigel implant animal model and histological analysis. MB assisted in FACS analysis. VS participated in histological analysis and conceived the experiments. VT participated in the design of the study. YP participated in the design of the study, its coordination and helped to draft the manuscript. All authors read and approved the final manuscript.
